# Evaluation of a Model for Glycemic Prediction in Critically Ill Surgical Patients

**DOI:** 10.1371/journal.pone.0069475

**Published:** 2013-07-19

**Authors:** Scott M. Pappada, Brent D. Cameron, David B. Tulman, Raymond E. Bourey, Marilyn J. Borst, William Olorunto, Sergio D. Bergese, David C. Evans, Stanislaw P. A. Stawicki, Thomas J. Papadimos

**Affiliations:** 1 Department of Bioengineering, University of Toledo, Toledo, Ohio, United States of America; 2 Center for Diabetes and Endocrine Research, University of Toledo, Toledo, Ohio, United States of America; 3 Department of Anesthesiology, The Ohio State University Wexner Medical Center, Columbus, Ohio, United States of America; 4 Division of Endocrinology, University of Toledo, College of Medicine, Toledo, Ohio, United States of America; 5 Department of Surgery, University of Toledo, College of Medicine, Toledo, Ohio, United States of America; 6 Department of Surgery, The Ohio State University Wexner Medical Center, Columbus, Ohio, United States of America; University of Michigan Medical School, United States of America

## Abstract

We evaluated a neural network model for prediction of glucose in critically ill trauma and post-operative cardiothoracic surgical patients. A prospective, feasibility trial evaluating a continuous glucose-monitoring device was performed. After institutional review board approval, clinical data from all consenting surgical intensive care unit patients were converted to an electronic format using novel software. This data was utilized to develop and train a neural network model for real-time prediction of serum glucose concentration implementing a prediction horizon of 75 minutes. Glycemic data from 19 patients were used to “train” the neural network model. Subsequent real-time simulated testing was performed in 5 patients to whom the neural network model was naive. Performance of the model was evaluated by calculating the mean absolute difference percent (MAD%), Clarke Error Grid Analysis, and calculation of the percent of hypoglycemic (≤70 mg/dL), normoglycemic (>70 and <150 mg/dL), and hyperglycemic (≥150 mg/dL) values accurately predicted by the model; 9,405 data points were analyzed. The models successfully predicted trends in glucose in the 5 test patients. Clark Error Grid Analysis indicated that 100.0% of predictions were clinically acceptable with 87.3% and 12.7% of predicted values falling within regions A and B of the error grid respectively. Overall model error (MAD%) was 9.0% with respect to actual continuous glucose modeling data. Our model successfully predicted 96.7% and 53.6% of the normo- and hyperglycemic values respectively. No hypoglycemic events occurred in these patients. Use of neural network models for real-time prediction of glucose in the surgical intensive care unit setting offers healthcare providers potentially useful information which could facilitate optimization of glycemic control, patient safety, and improved care. Similar models can be implemented across a wider scale of biomedical variables to offer real-time optimization, training, and adaptation that increase predictive accuracy and performance of therapies.

## Introduction

Severe traumatic injury represents a significant injury burden for the human body. Previous research clearly associates significant physiologic stress and acute hyperglycemic spikes, with elevated glucose levels serving as a form of “physiologic barometer” [1]. It is not surprising, therefore, that acute hyperglycemia is present in 25% or more of severely injured patients [2]. If hyperglycemia is sustained, indicating ongoing metabolic stress and/or difficulty maintaining glycemic control, mortality and morbidity are significantly increased [3]–[5]. If admission glucose levels exceed 200 mg/dL in severely injured patients, their expected survival may be reduced by as much as 50% [3]. Persistence of hyperglycemia during the first 2 days post-trauma has been shown to further reduce survival [5] and hyperglycemia during this early post-trauma period is associated with a multitude of adverse outcomes [3]. Moreover, delayed treatment of hyperglycemia does not appear to improve these outcomes [2]. Aggressive therapy to maintain serum glucose levels below 150 mg/dL is associated with improved outcomes [6]. In fact, active glycemic management aimed at lowering glucose levels after severe trauma has been associated with a reduction in mortality, length of time on ventilators, incidence of infection, and length of stays in the intensive care unit and hospital. Unfortunately, this goal can be elusive in trauma patients that require multiple critical care therapies [4]. In addition to maintaining serum glucoses within the relatively narrow therapeutic window described above, it is important to note that glycemic variability also plays an important role as a predictor of survival in critically ill surgical patients, with glycemic variability among non-survivors being twice the variability of survivors [1].

Appropriate glycemic control (glucose <180 mg/dL) in cardiac surgical patients also correlates with reduced morbidity and mortality [7]–[12]. Although the need for accurate glycemic prediction is clear, especially when optimizing glycemic control in critically ill surgical patients, the area of predictive modeling continues to receive little attention.

In this context, real-time continuous glucose monitoring (rtCGM) represents an important element in the overall aggressive approach to treatment of hyperglycemia. At a specified range of time horizons, rtCGM facilitates the assessment of trends in glycemic excursions over time. Utilization of rtCGM in critically ill patients has only recently gained increasing attention as a topic of active clinical research [13]. In a recent study, rtCGM utilized in an intensive care setting did not appear to result in improved outcomes [14]. However, rtCGM was noted to be especially useful in the context of detection and treatment of hypoglycemia. Importantly, rtCGM in the aforementioned investigation was utilized as only a monitoring tool. We hypothesize that the utilization of rtCGM in critical care patients combined with a system capable of predicting hyper- and hypoglycemia may enhance glycemic control, reduce glycemic variability, and consequently result in improved patient outcomes [6], [15]–[17].

The objective of this study was to evaluate the feasibility of utilizing a neural network model (NNM) for glycemic predictions across a broad range of critically ill surgical patients. The current NNM was configured to forecast projected glucose concentration 75 minutes into the future. Our NNM was integrated with CGM data and electronic medical records, reflecting real-time data acquisition throughout each patient’s intensive care stay. We hypothesized that a NNM configured to take into account diverse factors would demonstrate enhanced performance in comparison to previously developed and tested models.

## Materials and Methods

### Patient Recruitment and Enrollment Criteria

The study protocol was approved by the University of Toledo Institutional Review Board. Informed consent was obtained from all patients or their designated representative prior to enrollment. The consent was written and was approved by the Ethics Sub-committee of the Institutional Review Board. All consents had co-signature of an investigator and were secured using a double-lock system, i.e., all forms were locked in a secure cabinet that was locked in a secure room. The study population of this prospective feasibility trial consisted of patients admitted to the University of Toledo Medical Center surgical intensive care unit (SICU). Patients were recruited for study if they met the following criteria: trauma or cardiothoracic surgical intervention, glucose ≥150 mg/dL upon admission to the SICU, male or female aged ≥18 years. Cardiothoracic surgical patients previously diagnosed with type 1 or type 2 diabetes mellitus automatically met inclusion criteria. The only exclusion criterion was pregnancy. The trauma patients in the study had no documented history of diabetes, and the cardiac surgery patients all had documented type 2 diabetes. The model was not controlled for these differences.

### Data Acquisition and Collection

Upon study enrollment, a CGM device (Medtronic Diabetes, CGMS Ipro®) was placed subcutaneously to record interstitial glucose concentration measurements every five minutes during the course of each patient’s SICU stay. The CGM devices were calibrated (retrospectively prior to downloading CGM data) using POC blood glucose values measured through each patient’s length of stay in the SICU. The POC blood glucose meter used was an Accu-Chek Advantage manufactured by Roche Diagnostics and this glucose meter was used throughout the study.

**Figure 1:**
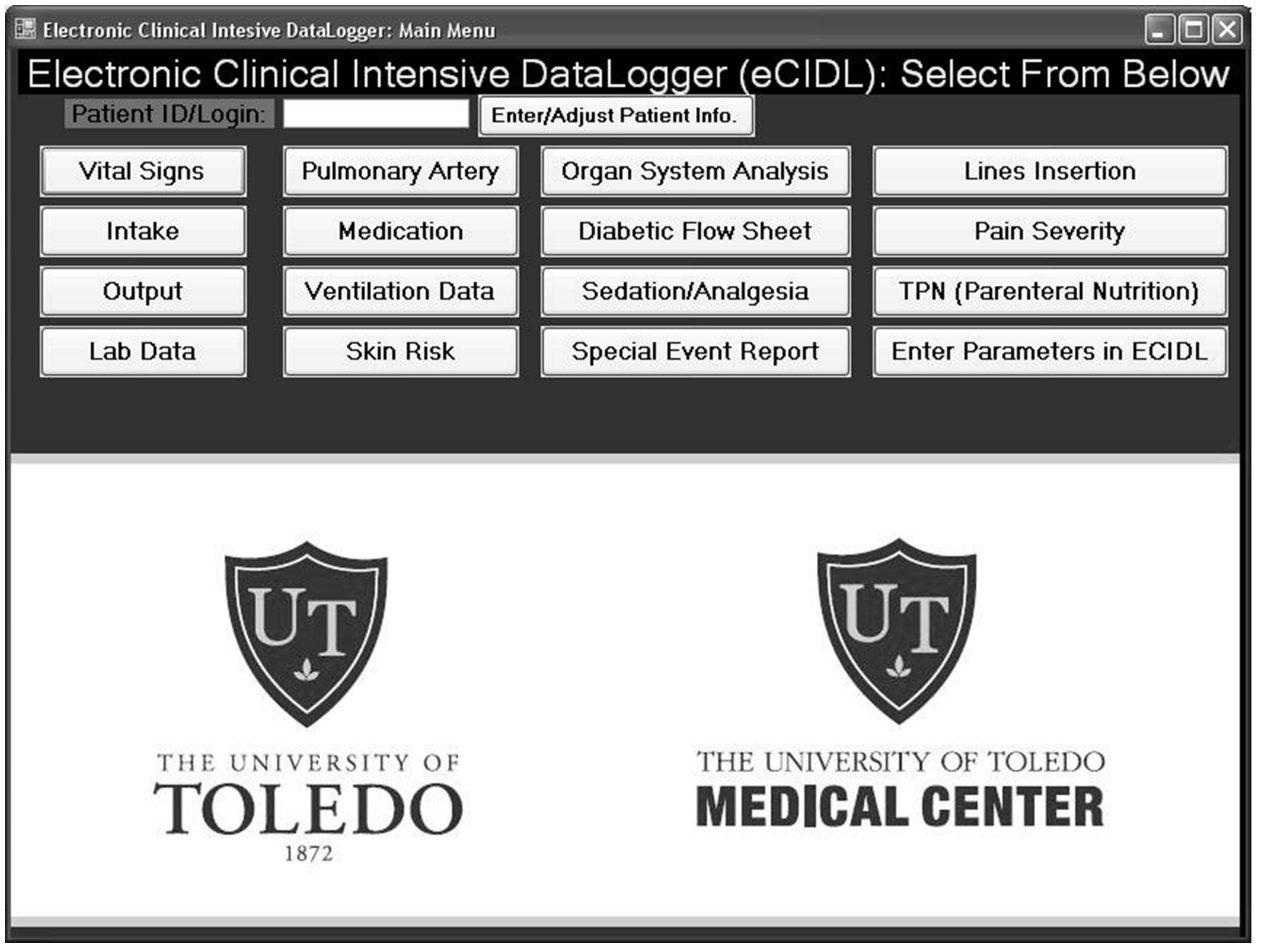
The main menu of the developed Electronic Clinical Intensive Data-Logger (eCIDL). This main menu contains buttons that link the user to various interfaces which contain text fields and drop-down menus to log all medical records present in the comprehensive intensive care unit medical record. This software application was utilized to convert paper-based medical records into electronic records suitable for direct neural network model utilization.

Using a custom computer program developed for this investigation, parameters in each patient’s paper-based medical records were converted to an electronic format. This data included all medical records collected at regular intervals during each patient’s SICU stay. The main menu of the computer program (Electronic Clinical Intensive Data Logger or eCIDL) is shown in **Figure 1**. From the main menu, buttons link the user to interfaces where medical records could be logged in a format suitable for use in NNM. The electronic medical records were categorized under 15 distinct categories, all found in Figure 1. The model was “trained” using data from 14 trauma and post-operative cardiothoracic surgical patients. Compared to our early models, the current NNM is more complex [18]. Our NNM was integrated with CGM data and electronic medical records, reflecting real-time data acquisition throughout each patient’s intensive care stay.

From the 15 categories listed in Figure 1, a total of 131 instantaneous distinct electronic medical records could be documented using the eCIDL software. This data was available for use in subsequent NNM development. To determine which of these medical records could be utilized as predictors of glucose concentration, a genetic algorithm (GA) was implemented. GAs, which are inspired by the principles of natural evolution, are useful techniques that have been utilized to optimize generation of useful solutions and to solve search related problems [19]–[21]. These algorithms are problem-solving techniques that use the evaluation of feedbacks (in computers) to improve performance. They belong to a larger set of evolutionary algorithms that can generate answers to problems of optimization using methods spurred by natural evolution (inheritance, mutation, crossover and selection). For an *x-block* of predictor data and *y-block* of data to be predicted, the key variables from the *x-block* can be identified in order to minimize the error in the *y-block* predictions. Here, through cross-validation and regression to determine the root mean squared error of cross validation, we obtained a subset of variables from the *x-block* are utilized for prediction. For this investigation, the *x-block* included the documented medical records, CGM device sensor current, and CGM values categorized as glycemic states. The *y-block* was defined as CGM data measured every five minutes.

### Neural Network Model Design and Development

**Figure 2:**
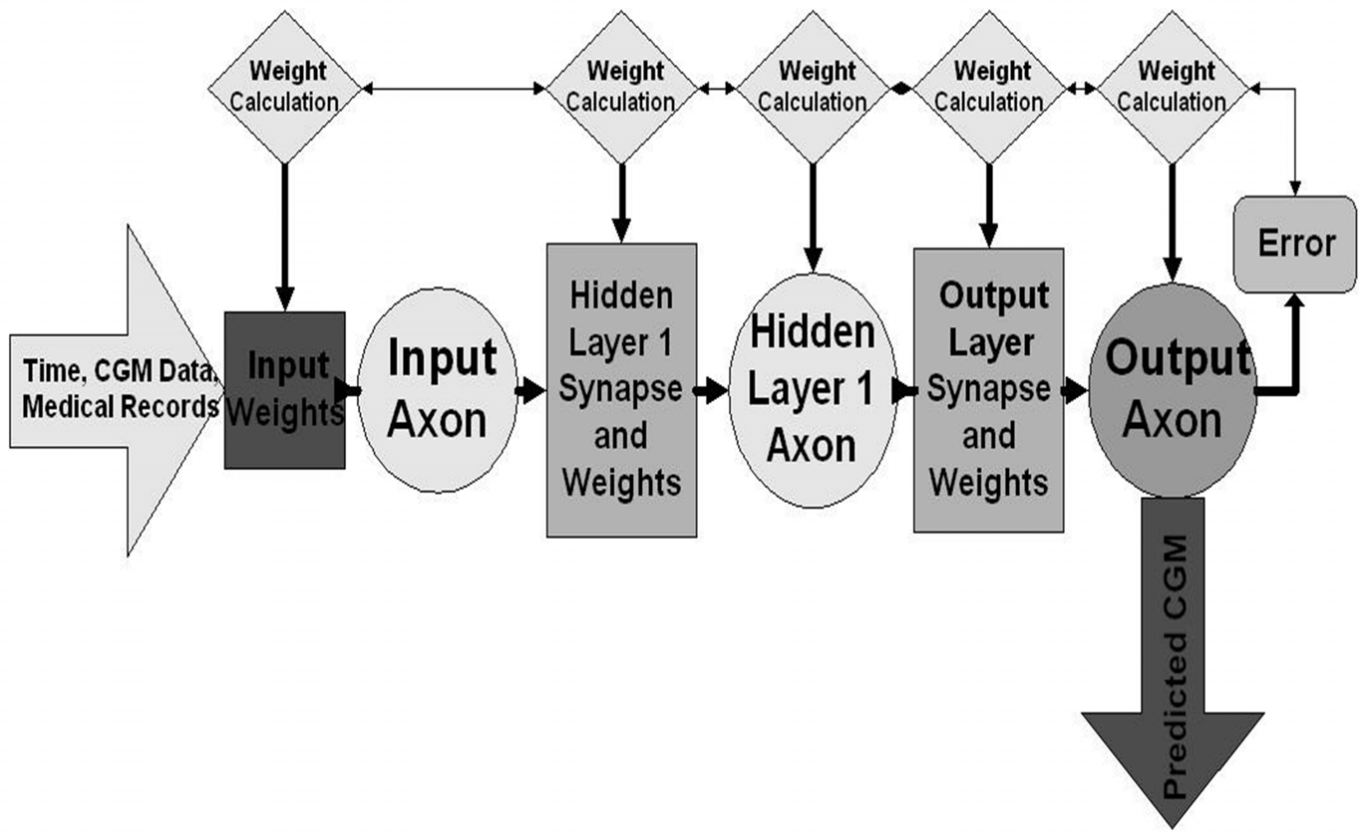
Neural network model design. The feed-forward neural network design implemented for real-time prediction of glucose. Error (mean squared error) is calculated between neural network output and desired response (actual continuous glucose monitoring values). This error is back propagated to each layer in the neural network architecture and a gradient descent with momentum algorithm is implemented to determine optimal weight values to minimize model error.

The neural network architecture included a three layer design consisting of a single input layer, a hidden layer for processing and an output layer; the NNM generated in this investigation was a feed-forward mechanism. **Figure 2** illustrates the NNM architecture and three layer design, and also demonstrates the flow of the data through the NNM, thereby decreasing computation time.

The NNM was developed using NeuroSolutions® software (Neurodimension, Gainesville, FL), and configured to predict glucose using a prediction horizon of 75 minutes. POC glucose readings in critical care patients with lack of tight glycemic control are generally obtained every 1–2 hours. This prediction horizon would provide insight into the time periods where POC glucose monitoring gaps. Implementation of models with prediction horizons >75 minutes may reduce accuracy and performance [22]. Additionally, there exists a reported time lag between interstitial and serum glucose concentration of 12.5 minutes [23], and our model mitigates the effects of this time lag.

The NNM was trained via a backpropagation is demonstrated in Figure 2. The error of the neural network predictions (mean squared error between actual and predicted CGM data) optimizes weights for minimization of error. The weight values that correspond to the smallest error are thus the optimal solution (set of NNM weights) for the particular NNM.

### Integration of Neural Network Model for Real-Time Prediction

The NNM functionality was integrated into a computer application for real-time prediction of glucose. Combined electronic medical records and CGM data from 5 of 19 patients consisting of 603 data points (50.3 hours of data not utilized for model training) were utilized for neural network model performance analysis.

For this investigation model weights were kept constant and not updated using the real-time application. To simulate real-time glycemic prediction, the real-time software application was configured such that every five minutes (sampling rate of the CGM device utilized in the investigation), a new real-time vector of variables from medical records and a CGM value was presented to the NNM and real-time prediction cycle was repeated.

### Performance Analysis of Real-Time Predictive Application

Model predictions were imported into MATLAB® (The MathWorks, Inc., Natick, MA) for performance analysis, which included the generation of a Clarke Error Grid to determine the clinical acceptability of the predictions, error calculation (mean absolute difference percent [MAD%]) between actual and predicted CGM values, and the percentage of normal, elevated, and low glucose predicted. Hyperglycemia was defined as glucose ≥150 mg/dL, hypoglycemia was defined as glucose ≤70 mg/dL, and normoglycemia was defined as glucose >70 and <150 mg/dL.

## Results

Data from 14 of 19 critical care patients that consisted of 19,989 input vectors (1,665.8 hours of data) was used for neural network model development and training. Of these 14 patients, 8 patients were trauma patients with average age of 49.5 years and average length of stay in intensive care of 9 days. Six patients were cardiothoracic surgical patients with average age of 75 years and average length of stay of 4 days in intensive care. The demographic characteristics of the enrolled patients used for predictive model development are detailed in Table 1. Overall, there was a higher proportion of male patients (*M = *10, 71.4%) than female. The overall patient population also exhibited a wide age range with a mean age of 59.3 years (*SD* = 18.1), and a mean BMI of 30.6 kg/m^2^ (*SD* = 4.7). As shown in Table 1, trauma and cardiothoracic patients differed in terms of age and BMI. Data from the remaining 5 of 19 patients was used to test the accuracy and clinical acceptability of the model. Of these 5 patients, 3 patients were trauma patients and 2 patients were cardiothoracic surgical patients.

**Table 1:** Patient Demographics.

N = 14	Trauma	Cardiothoracic Surgery	Overall
n	8	6	14
Male (%)	6 (75%)	4 (66.7%)	10 (71.4%)
Age (yr)*	47.7±14.9	74.6±6.4	59.3±18.1
BMI (kg/m^2^)*	33.1±3.6	27.7±4.2	30.6±4.7

This table includes the demographics of the patients enrolled in the study and used for model development. The patients are divided into two groups based on ICU admission type (trauma or cardiothoracic surgical intervention). Key demographics include: percentage of male patients, age, and BMI.

* Values presented as Mean ± SD.

Through implementation of the genetic algorithm, the number of model inputs was reduced by 69.4% to 40 inputs. Predictor variables included, but were not limited to, time of day (converted to a 24-hour scale), heart rate, respiratory rate, usage of intravenous dextrose solutions, point of care (POC) blood glucose measurements (and test times), units of insulin delivered, and insulin delivery type (intravascular infusion or subcutaneous sliding scale injections). Successful identification of predictor variables via implementation of the genetic algorithm was verified through literature review and search. For example, increased heart rate or tachycardia has been correlated in the literature with increased glucose concentration [24]. Furthermore, research has substantiated that body temperature is an indicator of glucose concentration specifically the occurrence of hypoglycemia [25]. Other factors/inputs such as dextrose solutions (D5, D5W, etc.) which are infused in critical care patients contain dextrose (glucose) which would correlate to an increase in glucose concentration. Therefore the optimized input set determined via implementation of the genetic algorithm coincides with literature review and discussion with clinical investigators. A complete listing of all 40 inputs utilized for model development can be found in reference [22].

The accuracy of current CGM technologies was also assessed via utilization of CEGA to compare CGM performance to that of blood glucose meters. Region A contains predicted values within 20% of the reference concentration and region B contains predictions outside 20% that would not lead to inappropriate treatment. Regions A and B therefore contain predicted values which can be classified as “clinically acceptable.” Region C contains points that lead to unnecessary treatments, and region D contains points indicating a potentially dangerous failure to detect hypoglycemia. Region E contains predicted values that confuse treatment of hypoglycemia for hyperglycemia and vice versa. A successful predictive model and system would thus need a majority of predicted CGM to fall within regions A and B of the Clarke Error Grid.

In order to ensure that the CGM device was providing accurate measurements, the error of reported CGM values with respect to POC blood glucose values was calculated. The error (MAD%) of CGM (interstitial glucose) values with respect to POC blood glucose values in the model training set was calculated as 10.2% for 995 paired CGM and blood glucose values. For this investigation, model predictions were compared against actual CGM values reported by the CGM device.

**Figure 3:**
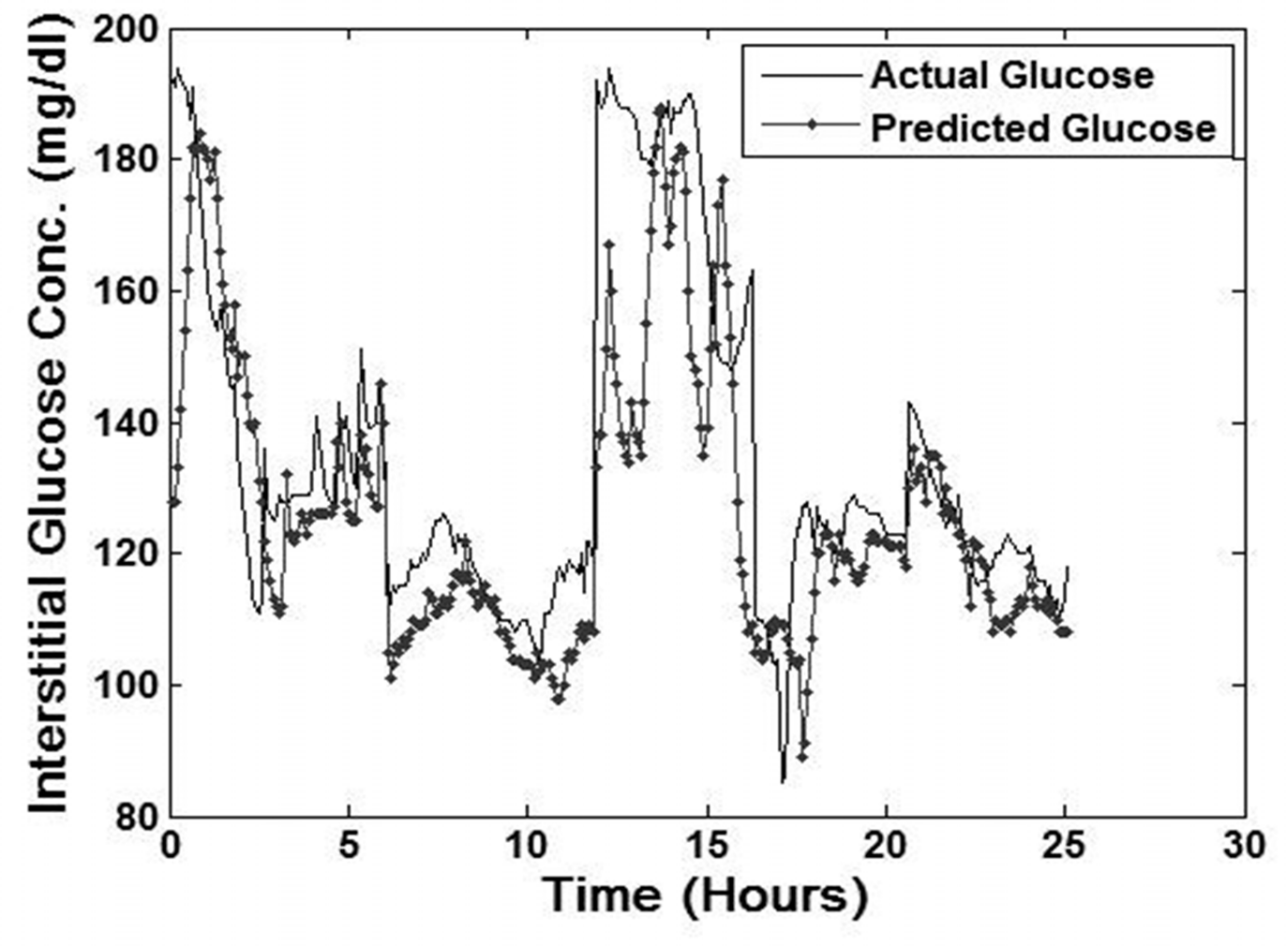
Glycemic predictions generated by neural network model.

Actual CGM and predicted CGM values generated by the real-time application in the five patients not utilized for model training are depicted in **Figure 3** **(**this graph represents sample predictions, not mean predictions, made across five patients not used for model training. We used only random segments of data to generate these predictions). Due to the large dataset of 9,405 predicted glucose values (derived from 15 CGM values recorded every 5 minutes for 75 minutes predicted for every CGM value in the test dataset) the data was re-sampled to demonstrate predictive accuracy. Re-sampling was completed via plotting every 20^th^ predicted CGM value and corresponding actual glucose value in the predictive dataset. In calculating overall error between actual and predicted CGM values, the MAD% was calculated as 9.0%. In this dataset, 96.7% of normal glucose values and 53.6% of hyperglycemic values were predicted. No hypoglycemia occurred in the patient data utilized for assessment of model performance.

**Figure 4:**
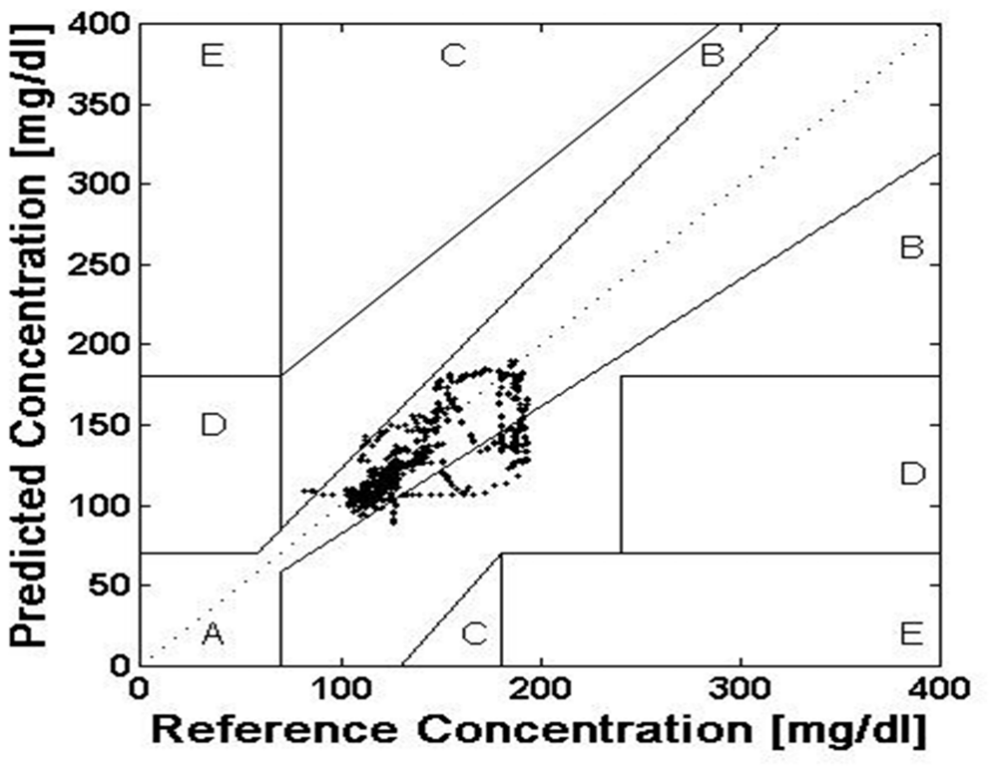
Clarke Error Grid Predictions demonstrating 97.5% clinically acceptable predictive values.

**Figure 4** contains Clarke Error Grid Analysis (CEGA) of real-time predictions in five critical care patients. In this dataset, 9,405 predicted values were generated. CEGA demonstrated 100.0% of predicted values were clinically acceptable with 87.3% and 12.7% falling within regions A and B of the error grid respectively. CEGA also indicated that 0.0% of predicted values were in regions C, D, and E, which would have resulted in inappropriate and potentially adverse therapy.

Real-time predictive accuracy of the model developed in this investigation expressed by overall error (MAD%) of 9.0%, falls within acceptable error limits as defined in previous reports [26]. Furthermore, 100.0% of predictions fell within regions A and B of the Clarke Error Grid. The test data set consisted of 23.4% hyperglycemic data and 76.6% normoglycemic data. No hypoglycemic data occurred in the 5 patient test dataset used for model performance analysis. The model successfully predicted 96.7% of normal glucose values and 53.6% of elevated glucose values. Predictive accuracy demonstrated in our investigation suggests that such models can provide useful information that can potentially facilitate intelligent therapeutic guidance and clinical decision support.

## Discussion

Therapy of hyperglycemia in critically ill patients at the University of Toledo Medical Center relied on POC blood glucose measurements obtained every 1–2 hours. Insulin is administered by continuous infusion or subcutaneous injection based on glucose-driven protocols. Although sampling of glucose concentrations via POC measurement provides a means to glycemic control, hypoglycemia or hyperglycemia can occur between glucose measurement and correction. Therefore, there is a need to know or predict glucose concentration during these time periods where healthcare providers are “blind” to the glycemic excursions and trends.

This study demonstrates that real-time prediction of glucose concentration in critical care patients is possible. In this patient population, prediction of glucose using a horizon of 75 minutes may provide information to prevent unacceptable glycemic excursions. Prediction of high or low glucose levels would allow caregivers to modify insulin infusion rates and, when applicable, administer exogenous dextrose or glucose to avoid hypoglycemia, thus further optimizing glycemic control.

Real-time predictive paradigms require data input systems with high degree of accuracy. There are established methods to determine accuracy and quantify the degree of “clinical acceptability” of model predictions. It was previously reported that the error of CGM devices with respect to “gold standard” serum glucose values ranges from 14–21% [24]. An acceptable forecasting model of CGM values should therefore be characterized by similar or better predictive error rates. Another method of analyzing the clinical acceptability of predictive models is through Clarke Error Grid Analysis CEGA. CEGA was described in 1987 and was originally utilized to assess meter-based patient estimates of blood glucose compared to those obtained using a “gold-standard” reference glucose meter [27], [28].

Although no hypoglycemic events occurred in the data from the 5 critical care patients used to test model performance, we hypothesize the model may overestimate hypoglycemic extremes. The initial model training set composition included 4.5%, 11.7%, and 83.8% hypo-, hyper- and normoglycemic states respectively. We speculate that the current model overestimates hypoglycemia based on the lack of a significant quantity of hypoglycemic training data. Model training set composition is highly associated with model performance [29]. Our previous research demonstrated that similar model training sets with reduced quantities of hypoglycemic training data would lead to overestimation of hypoglycemic extremes [30].

The models generated in this investigation also have a reduced ability to predict hyperglycemia. In this investigation, 53.6% of hyperglycemia was predicted successfully. This finding can also be attributed to the percentage of hyperglycemic training data (11.7%) in the comprehensive model training set. The model performs with high accuracy in predicting normal glycemic values, which correlates with the large quantity of normal training data (83.8%). Further data acquisition is ongoing and will be a focus of future research such that training sets with larger quantities of hypoglycemic and hyperglycemic extremes are achieved.

Variations in glycemic patterns characterize the clinical course of most patients in the critical care setting [31]. However, prediction of glucose remains challenging. Hemodynamics, medications, laboratory results, and nutritional intake are potential modulators of future glucose concentration. A successful model for prediction of glucose in the critically ill patient population would need to consider and incorporate real-time influences of numerous variables that participate in modulation of serum glucose values. An approach based on NNM is well suited to characterize such a complex system in which numerous factors are predictive of glycemic excursions. NNMs can be categorized as non-linear data-modeling and/or decision-making tools. These systems actively adapt their structure based on external or internal information as they are processed.

NNM systems have been studied as a predictive tool in the area of maintaining desired glucose concentrations using glucose and/or insulin dosing algorithms in patients with diabetes [32]–[35]. One downside to NNMs utilized in previously published studies was the fact that they were based on discrete blood glucose measurements obtained by portable glucose meters. Additional limitations to early NNMs include failure to incorporate additional variables important to glucose prediction such as circadian cycles, work, operating a motor vehicle, stress, and depression [36]–[40]. To this end, we previously developed an enhanced NNM, which accurately predicted glucose between 50–180 minutes ahead of time in patients with insulin-dependent diabetes [41]. This modeling application was among the first NNMs to integrate CGM and record multiple metabolic events and activities to predict future glycemic excursions. Subsequently, modeling techniques utilizing similar approaches have attracted significant interest [42].

NNM systems also have been investigated for the potential to predict glucose and/or optimize glycemic control in the critical care setting [43]. Many of these modeling approaches have utilized only conventional inputs that include discrete POC blood glucose values, insulin infusion data, and nutritional intake. Our group has investigated the potential of utilizing patient-specific NNMs for prediction of glucose in the surgical critical care setting [18]. During these initial studies, we developed simplified NNMs that utilized a limited number of relevant input variables, including POC blood glucose determination, CGM, and insulin delivery data for glycemic prediction.

The NNMs, as generated in this investigation for prediction of glucose, used model weights obtained via comprehensive model training. A key feature of NNMs is the ability to adapt model weights based on the real-time occurrence of input data presented to the network to minimize model predictive error. Ongoing research aims to optimize the real-time application utilized in this investigation to provide real-time model training and weight adaptation within the sampling rate of the CGM device. Given this capability, when medical records and CGM data are acquired in real-time, model weights will be updated such that maximal predictive accuracy is achieved. Theoretically, models implementing real-time/on-line training will have significant increases in performance with respect to the model developed in this investigation.

Given that acute glycemic excursions have been correlated with adverse clinical events [1], it is reasonable to speculate that models integrating NNM-based glycemic prediction and indicator-based system of “clinical alerts” could further enhance the armamentarium of clinical predictive tools available to the intensivist. Such integrated systems could utilize existing CGM and POC glycemic testing, not only to alert the bedside practitioner to potential glycemic dysregulation within a near-term horizon, but also to alert the critical care team to look for potential underlying events that may incite such glycemic excursions. Further advantages of utilizing complex NNM-based glycemic prediction systems include the potential to integrate additional variables that may indirectly reflect worsening glycemic hemostasis, such as gradual increases in insulin drip requirements or escalating need for exogenous glucose administration.

Several further criticisms of our trial must be noted. We have defined this study as a feasibility trial because of the small numbers used to train the model (n = 14) and to test it (n = 5). The lack of separation between those with stress hyperglycemia and diabetes diminish our model when the sample size is as small as it was in this trial. This lack of differentiation between the groups may be the reason our model poorly predicted hyperglycemia at rates greater than 54%. Hypoglycemia prediction was a problem since the patient data was lacking for this. Therefore, the model could be criticized as less robust a predictor of anything but normal blood glucose values.

Despite these shortcomings this work demonstrates the feasibility of using neural network modeling for glycemic prediction in the critically ill patient, which has not only potential for physiologic, morbidity and mortality benefits, but also economic benefits.

Further research should include the development of post-processing algorithms to modify neural network model output given the occurrence of input data (medical records and CGM data) in real-time. Factors such as tachycardia, medications, and real-time trends in CGM data may successfully predict future glycemic trends. A successful model will be able to account for such interdependencies, such as medications that increase insulin resistance or high blood glucose concentration (which functions to slow gastric glucose absorption). The analysis and modeling of glycemic responses following such events might provide means for further development of post-processing algorithms to enhance neural network model performance.

### Conclusion

Real-time application of a CGM-based NNM for glycemic prediction in critically ill surgical patients is feasible. Knowledge of predicted glucose values may provide a means to improve and better optimize glycemic control in patients at risk for development of rapid hypo- or hyperglycemic oscillations. The results of this investigation indicate that a model trained using data from multiple subjects can provide predictive accuracy with a better-than-expected degree of clinical acceptability across a diverse patient base. Future research investigating whether significant increases in model performance can be achieved via implementation of real-time/on-line training is warranted.

In the future, model predictions may be able to facilitate intelligent therapeutic guidance/direction and clinical decision support. When placed in the appropriate context, prediction of glucose concentration would provide caregivers a means preemptively to modify therapy and clinical interventions in order to improve glycemic control. Optimization of glycemic control in critical care patients would result in significant improvements in patient safety, care, and outcome. While CGMS alone can provide an alert to medical providers, it cannot do so 75 minutes out. Therefore, the addition of a predictive clinical alert systems based on parallel, multi-indicator processing algorithms may help alert the bedside clinician not only to glycemic oscillations but also to the possibility of untoward clinical events that may be underlying the “barometer-like” behavior of glucose.
